# Prevalence of Adverse Reactions to Glutenand People Going on a Gluten-Free Diet:A Survey Study Conducted in Brazil

**DOI:** 10.3390/medicina56040163

**Published:** 2020-04-04

**Authors:** Jesús Gilberto Arámburo-Gálvez, Carlos Eduardo Beltrán-Cárdenas, Tatiane Geralda André, Itallo Carvalho Gomes, María Auxiliadora Macêdo-Callou, Élida Mara Braga-Rocha, Elaine Aparecida Mye-Takamatu-Watanabe, Vivian Rahmeier-Fietz, Oscar Gerardo Figueroa-Salcido, Marcela de Jesus Vergara-Jiménez, Lilian Karem Flores-Mendoza, Noé Ontiveros, Francisco Cabrera-Chávez

**Affiliations:** 1 Postgraduate in Health Sciences, Division of Biological and Health Sciences, University of Sonora, Hermosillo, Sonora 83000, Mexico; gilberto.aramburo.g@gmail.com (J.G.A.-G.); gerardofs95@hotmail.com (O.G.F.-S.); 2 Faculty of Nutrition Sciences, University of Sinaloa, Culiacán, Sinaloa 80019, Mexico; carlos.1.beltran@hotmail.com (C.E.B.-C.); mjvergara@uas.edu.mx (M.d.J.V.-J.); 3 Master of Science Program in Nursing, School of Nursing, Los Mochis, Sinaloa 81220, Mexico; tatianegrandre@gmail.com (T.G.A.); carvalhoitallo@gmail.com (I.C.G.); 4 Faculdade de Juazeiro do Norte, Juazeiro do Norte, Ceará 63010-215, Brazil; auxiliadora.callou@fjn.edu.br (M.A.M.-C.); elidamara@usp.br (É.M.B.-R.); 5 Curso de Enfermagem, Universidade Estadual de Mato Grosso do Sul, Dourados, Mato Grosso do Sul 79804-970, Brazil; swatanab@terra.com.br (E.A.M.-T.-W.); vivian@uems.br (V.R.-F.); 6 Department of Chemical, Biological, and Agricultural Sciences (DC-QB), Division of Sciences and Engineering, Clinical and Research Laboratory (LACIUS, URS), University of Sonora, Navojoa, Sonora 85880, Mexico; lilian.flores@unison.mx

**Keywords:** celiac disease, NCGS, wheat allergy, gluten-free diet, self-report, survey studies, gluten-related disorders

## Abstract

Background: The prevalence of gluten-related disorders (GRD) and adherence to a gluten-free diet (GFD) remains unknown in Brazilian population and there is no published information on the scientific literature about the proportion of Brazilians that were diagnosed with a gluten-related disorder. Thus, the aim of this work was to estimate the prevalence of GRDs and adherence to a GFD by self-report in adult Brazilian population. Materials and Methods: A questionnaire-based cross-sectional study was conducted in two Brazilian cities. Results: The response rate was 93.2% (1630/1749). The self-reported prevalence rates were (95% CI): adverse reactions to gluten 10.06% (8.64–11.62); gluten sensitivity 2.33% (1.65–3.18); physician-diagnosed celiac disease 0.3% (0.09–0.71); non-celiac gluten sensitivity 1.71% (1.14–2.47); wheat allergy 0.79% (0.42–1.36); adherence to gluten-free diet 7.48% (6.25–8.87); gluten avoiders 15.21% (13.5–17.05). Among those who were following a GFD (*n* = 122), 65.6% (*n* = 80) of them reported that they did not develop symptoms after wheat/gluten ingestion and 50% (*n* = 61) were following the diet without medical/dietitian advice. The main motivation for following a GFD in the self-reported and non-self-reported gluten sensitivity groups were the symptoms triggered after wheat/gluten ingestion (86.8%) and weight control (57.1%), respectively. Conclusions: Implementation of programs to increase awareness about GRDs among healthcare professionals and giving scientifically sound information to the general population about the risks and benefits for following a GFD are desirable actions in Brazil. The results also add to the growing body of evidence for highlighting the under-diagnosis of GRD and the trend for following a GFD in Latin America.

## 1. Introduction

Disorders triggered by wheat/gluten include celiac disease (CD), which is also triggered by gluten from rye and barley, wheat allergy (WA) and non-celiac gluten sensitivity (NCGS). CD is a T cell-mediated autoimmune-like enteropathy associated with inflammation. The hallmark of IgE-mediated WA is the presence of anti-wheat IgE antibodies in patients’ blood with the appearance of symptoms after wheat exposition [1]. NCGS has a wide spectrum of clinical manifestations that commonly overlap with irritable bowel syndrome and CD [2,3]. However, NCGS pathogenesis is still unclear and there is a lack of sensible and reproducible biomarkers to aid in its diagnosis [4]. Although experts have proposed criteria for NCGS diagnosis [5], some gaps need to be filled before using the criteria in clinical practice [6,7]. In this frame, the survey-based estimation of NCGS prevalence at population level has gained attention among researchers in the field. Additionally, the survey-based tools developed for such a purpose have allowed us to assess the observance of a dietary regime that avoids wheat/gluten (gluten-free diet, GFD). Adhering to a GFD or avoiding wheat from the diet (in WA cases) are the only accepted treatments for gluten-related disorders (GRD) [8]. However, most people following the GFD are doing it for reasons other than health related benefits and without medical/dietitian advice probably compromising their health [9,10,11,12]. In the last years, our research group has been perfecting and applying a tool in different Hispano American countries to estimate the prevalence rates of adverse reactions to gluten and adherence to a GFD [9,10,13,14]. Based on specific definitions, the prevalence rates of self-reported GRDs can be estimated. Recently, the tool was validated in Brazilian-Portuguese [15], opening the opportunity for carrying out a survey study in a country inhabited by almost half of the South American population. Thus, our aim was to estimate the prevalence rates of adverse reactions to gluten, of the disorders triggered by wheat/gluten ingestion and of following a GFD in two populations from Brazil.

## 2. Materials and Methods

### 2.1. Survey Tool and Data Collection

The questionnaire was validated in Brazilian-Portuguese [15]. Individuals who reported adverse reactions to gluten answered questions from the first section of the questionnaire. All the other participants answered questions from the second section of the questionnaire. All participants provided information about demographic data, adherence to a GFD, motivations to follow the diet and who instructs the diet. 

The data were collected in public places of two Brazilian cities (Juazeiro do Norte, Ceará and Dourados, Mato Grosso do Sul, Brazil) during the months of July and August of 2019. Participants were approached in urban parks and outside shopping malls and supermarkets located in the cities of Juazeiro do Norte and Dourados cities. Inclusion criteria were as follows: (1) Brazilian individuals who agreed to sign the informed consent; (2) ≥18 years old; and (3) subjects that were able to read and answer the questionnaire by themselves. Questionnaires with incomplete demographic data, such as age and gender, were excluded from the study. The interviewers (health sciences students) helped the interviewees when it was needed.

### 2.2. Criteria Used to Classify Adverse Reactions to Gluten and GRDs

**Figure 1:**
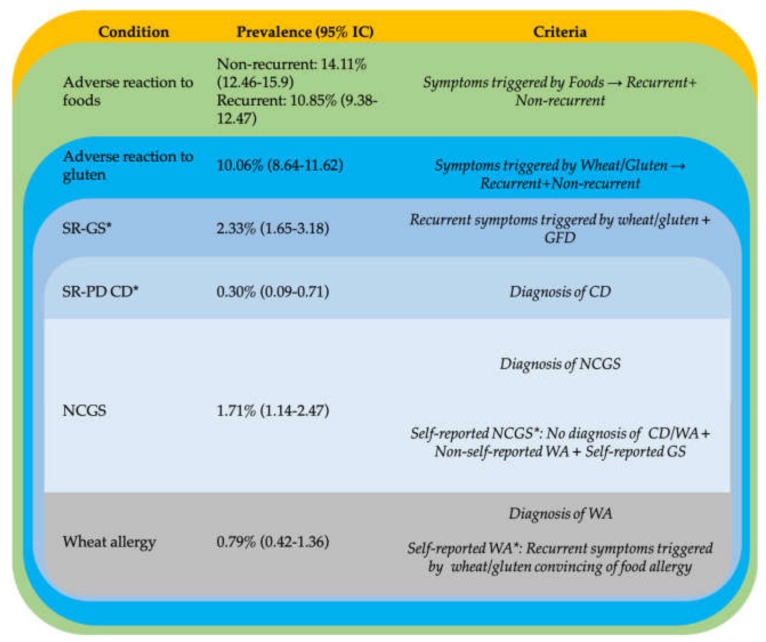
Definitions and prevalence rates estimations of adverse reactions to food and disorders triggered by wheat/gluten.

All criteria used to classify individuals in one or another condition were stated previously [9,10,13,14,15] (Figure 1; Table S1). The definitions given include the following terms: adverse reactions to foods, adverse reactions to gluten, self-reported gluten sensitivity (SR-GS), self-reported physician-diagnosed CD (SR-PD CD), self-reported physician-diagnosed WA (SR-PD WA), self-reported WA, self-reported physician-diagnosed NCGS and self-reported NCGS.

### 2.3. Ethical and Statistical Aspects

The survey was approved by two Ethic Committees: one from the Faculty of Juazeiro do Norte (NP: 3.382.689; date of ethic approval: 11 June 2019) and another from the Mato Grosso do Sul State University (NP: 3.443.878; date of ethic approval: 8 July 2019). Descriptive statistics (total numbers, percentages, odds ratio and 95% confidence interval) were used for categorical variables. Two-tailed Fisher’s exact test and Student t-test were applied to determine associations and mean differences, respectively, using the software GraphPad Prism Version 5.0 (GraphPad Software, San Diego, CA, USA). For statistical hypotesis tests, *p*-values lower than 0.05 were considered significant. The free version of OpenEpi software (3.01, www.OpenEpi.com, updated 6 April 2013) was used to calculate the prevalence rates, which were reported as a percentage (95% confidence intervals).

## 3. Results

### 3.1. Participation and Interviewees’ Characteristics

In this study, 1749 individuals were approached. The response rate was 93.2% (*n* = 1654). Twenty-four individuals were excluded of the study because they provided incomplete data. Therefore, 1630 documents were included in the study. Of the participants, 45.0% and 55.0% were male and female, respectively. Non-food allergy (7.91%), Lactose Intolerance (7.06%) and Diabetes Mellitus (3.87%) were the most commonly informed physician-diagnosed conditions. The individuals with SR-GS showed a significant association with irritable bowel syndrome (Odds Ratio, 95% CI, 5.78 (2.3–14.4)), psychiatric disease (Odds Ratio, 95% CI, 1.46 (0.34–6.25)), eating disorders (Odds Ratio, 95% CI, 7.98 (1.7–37.3)), autoimmune disease (Odds Ratio, 95% CI, 2.18 (0.65–7.31)) and lactose intolerance (Odds Ratio, 95% CI, 4.35 (2.0–9.4)). 

### 3.2. Proportions Estimations

The general prevalence of adverse reactions to wheat/gluten was 10.06% (*n* = 164) (Figure 1). In this group, 7.36% (*n* = 120) and 2.69% (*n* = 44) reported non-recurrent and recurrent adverse reactions to gluten, respectively, but only 38 individuals met criteria for SR-GS (General prevalence (95% IC), 2.33% (1.65–3.18)). Five cases meet criteria for physician diagnosis of CD (General prevalence (95% IC), 0.30% (0.09–0.71)) although one of these cases was on a regular diet. Comparisons between women and men revealed that the prevalence rates of adverse reactions to wheat/gluten (11.94 vs. 7.76%), recurrent adverse reactions to food (12.38 vs. 8.99%) and gluten avoiders (16.6 vs. 12.94%), but not other parameters assessed (*p* > 0.05) (data not shown), were higher in women than in men (*p* < 0.05).

**Figure 2:**
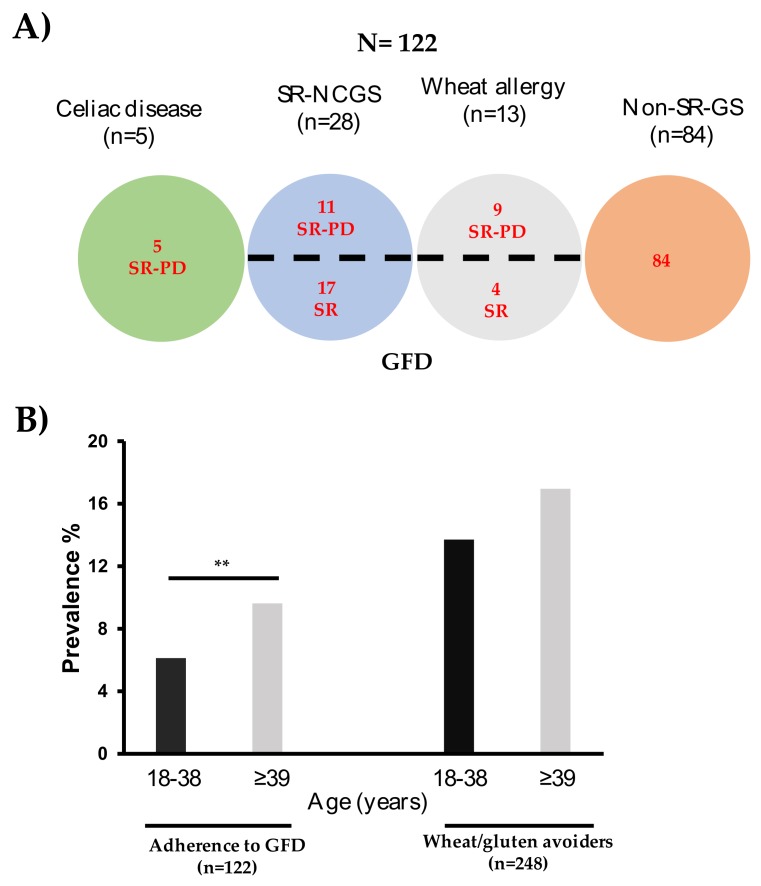
Individuals on a gluten-free diet (GFD) and wheat/gluten avoiders. (**A**) Characteristics of individuals following a GFD. (**B**) Adherence to a GFD and wheat/gluten avoiders stratified by age (Black bars: 18–38 years old, Grey bars: ≥39 years old).

The prevalence of adherence to a GFD was 7.48% (*n* = 122) (95% IC, 6.25–8.87). Notably, most people who were following a GFD were non-SR-GS cases (68.8%; *n* = 84) (Figure 2A). Among these, 95.2% (*n* = 80) did not report any adverse reactions to gluten and the other 4.8% (*n* = 4) reported non-recurrent adverse reactions to gluten. Among those who reported adverse reactions to oral wheat/gluten (*n* = 164), only 23.17% (*n* = 38) reported both recurrent adverse reactions to wheat/gluten and to be following a GFD. The prevalence of gluten avoiders was 15.21% (*n* = 248) (95% IC, 13.5–17.05) and most of the cases did not report any adverse reactions to gluten (79.4%; *n* = 197). Among the other 20.6% (*n* = 51) of the cases, 50 cases reported non-recurrent adverse reactions to gluten and only 1 case reported recurrent adverse reactions to gluten.

The proportion of subjects who were adhering to a GFD was larger in the group aged ≥39 than in the one aged 18–38 years old (9.59% vs. 6.14%; *p* = 0.01) (Figure 2B). A similar trend was observed in the group of gluten avoiders (*p* > 0.05) (Figure 2B).

**Figure 3:**
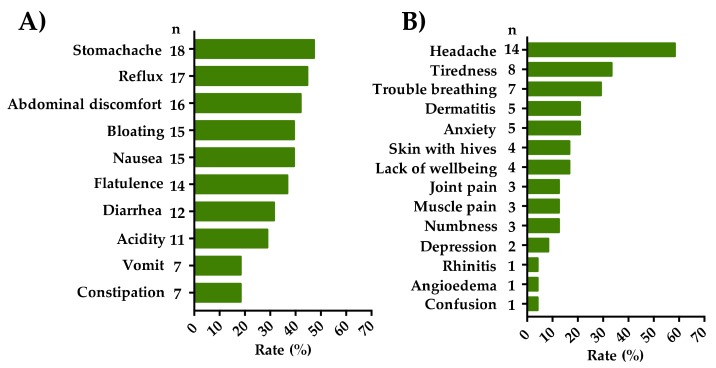
Recurrent self-reported gastrointestinal (part (**A**)) and extra-intestinal (part (**B**)) symptoms in self-reported gluten sensitivity (SR-GS) individuals.

All individuals that met criteria for SR-GS (*n* = 38) reported recurrent gastrointestinal symptoms triggered after wheat/gluten ingestion. In this group, 24 cases reported extra-intestinal symptoms also. At gastrointestinal level, stomachache (47.3%), reflux (44.7%) and abdominal discomfort (42.1%) were the main symptoms reported (Figure 3A). At extra-intestinal level, headache (58.3%), tiredness (33.3%) and trouble breathing (29.1%) were the most common symptoms (Figure 3B). Most individuals reported more than one symptom, either gastrointestinal or extra-intestinal, or both.

### 3.3. Reasons for Adhering to a GFD

**Figure 4:**
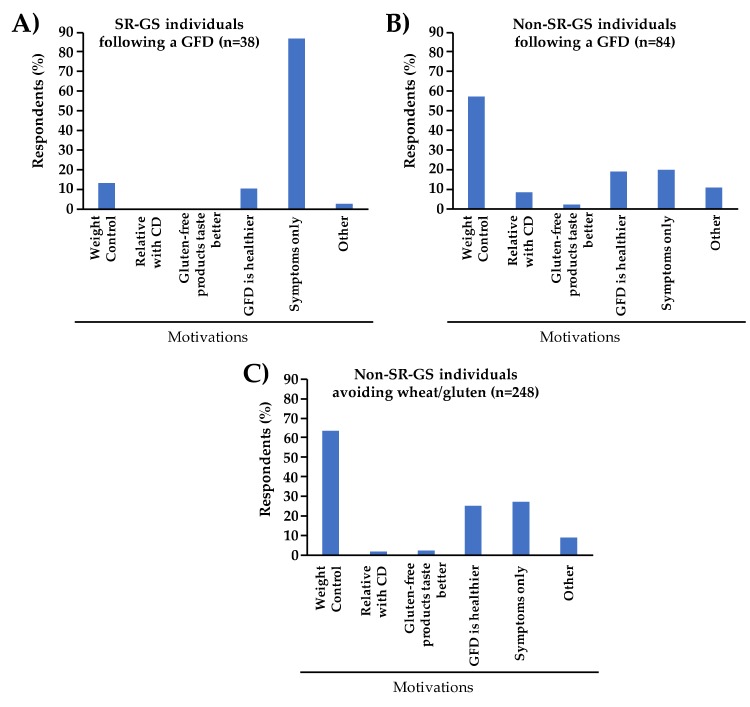
Motivations of SR-GS, and non-SR-GS cases for following a GFD (parts (**A**) and (**B**)) or avoiding wheat/gluten from their diets (part (**C**)).

A total of 122 individuals reported that they were adhering to a GFD. Among these, 38 met criteria for SR-GS and 84 were non-SR-GS cases. Regarding who instructed the GFD, 16 (42.1%) out of 38 SR-GS cases and 45 (53.6%) out of 84 non-SR-GS cases reported that they were following a GFD without medical/dietitian advice. In the SR-GS group (*n* = 38), the main motivation reported for following a GFD was the symptoms associated to wheat/gluten intake (86.8%) (Figure 4A). Other motivations, such as gluten-free products taste better and having a relative with CD, were not reported in this group. Contrary, in the non-SR-GS group, the main motivation reported for adhering to a GFD was weight control (57.1%) (Figure 4B). Similarly, among the wheat/gluten avoiders (*n* = 248), 63.5% reported weight control as the main motivation for following a GFD (Figure 4C).

## 4. Discussion

The prevalence rates of GRDs and adherence to a GFD in adult Brazilian population were estimated by self-report. Other survey studies with the same design and carried out in Latin America by using the same instrument have reported lower response rates (53%–92%) than the rate obtained in the present study (93.2%) [9,10,13,14]. It should be noted that this rate is ten-fold higher than the one reported in a pilot study carried out in Brazilian population, but an online platform was used to collect data [15]. Online surveys are practical and economical mainly because they do not require trained human resources moving from one place to another. Unfortunately, our results confirm that the instrument utilized in the present study requires a survey design involving face-to-face interviews for obtaining high response rates.

Although attention should be paid to the definitions given to a specific GRD in particular studies, the pooled prevalence of self-reported GRD among studies carried in Latin America (3.1%–7.8%) [9,10,13,14], Europe (6.2%–13%) [16,17,18,19,20] and Australia (14.9%) [21] range from 3.1% to 14.9%. The consumption of wheat has been related to the prevalence of GRD such as CD and/or NCGS [9,22], the higher wheat/gluten consumption per capita the higher prevalence of GRD expected, but this notion seems not to apply for the Brazilian populations studied. Brazilians consume more wheat per capita (59.9 kg) than Mexicans (55.2 kg), Colombians (29.48 kg) and Salvadorans (34.34 kg) [23,24,25,26], but the pooled self-reported prevalence of GRD was 2.33% in the present study. Certainly, this is the lowest prevalence rate of GRD ever reported among survey-based studies carried out in Latin American countries and elsewhere [9,10,13,14]. Despite these findings, the main gastrointestinal and extra-intestinal symptoms informed by those who met criteria for SR-GS were the same as those reported in different populations [9,10,13,14] including a Brazilian population suspected of NCGS [27]. Overall, these data highlight the need for further population-based epidemiological studies preferentially including serology tests, HLA-typing (Human Leucocyte Antigen-typing), intestinal biopsies and an in-depth questionnaire.

The prevalence rate of SR-PD CD was 0.30%. This prevalence rate is even lower than that reported in other studies carried out in a Brazilian population (0.36%), which were based on biopsy-proven CD [28]. Considering a general CD prevalence among populations between 0.5 and 1.0%, the results show a potential CD underdiagnosis in the Brazilian population. This potential underdiagnosis seems to be a common problem in some Latin American populations; for instance, among more than 1200 people surveyed in Mexico only 1 person met criteria for SR-PD CD [13]. Similar findings were reported in studies carried out in El Salvador (2 SR-PD CD cases in 1326 people surveyed) [10] and Colombia (no SR-PD CD cases in 1207 people surveyed) [14]. Contrarily, a study carried out in Argentina reported a prevalence rate of SR-PD CD of 0.58% (7 cases in 1209 people surveyed) [9]. This increase in CD diagnosis was attributed to the implementation of a nationwide program for the detection and control of CD and the subsidies given to the patients to help manage the cost of the GFD [9]. No program or subsidies have been implemented in Brazil. Therefore, the estimated prevalence rate of SR-PD CD in the Brazilian adult population surveyed in this study shows that awareness of CD by Brazilians health professionals is better than in some other Latin American countries.

NCGS diagnosis is challenging due to the non-standardized and time-consuming diagnostic criteria as well as the lack of sensitive and reproducible biomarkers. In the present study, the self-reported NCGS prevalence rate was estimated including people that met criteria for SR-GS, but did not meet criteria for WA or CD [9,10]. Under these criteria, the prevalence of NCGS in the Brazilian population studied was 1.7%. Utilizing the same criteria, this rate is higher than those reported in survey studies carried out in Mexico (0.16%), Colombia (0.82%), Argentina (0.91%), El Salvador (0.98%), The Unites States of America (0.54%) and Italy (slightly higher than 1%) [9,10,13,14,16,21]. It should be noted that the NCGS prevalence rate reported in the present study is the highest among the populations evaluated utilizing the same criteria, but, paradoxically, the pooled prevalence of GRD is the lowest. As mentioned above, further studies including celiac serology and HLA typing will help to identify the potential CD cases that in survey studies meet criteria for NCGS. Regarding WA, the prevalence rate was 0.79%. This rate is in line with WA prevalence data estimated in other surveys carried out in Latin American countries (Mexico, Colombia, Argentina, El Salvador; 0.72, 0.74, 0.33, 0.75%, respectively) and utilizing the same instrument [9,10,13,14]. The prevalence of food allergy, including wheat allergy (0.6%), in Brazilian infants was estimated by parent-report [29], but, to our knowledge, this is the first study that estimate the prevalence of WA in Brazilian adult population.

Four out of five SR-PD CD cases identified in the present study informed that they were following a GFD. In other studies around 60% of the Brazilian physician-diagnosed CD patients informed to be following a strict GFD [30,31]. This difference in the percentages of adherence to a GFD can be attributed to the targeted populations of each study and the number of physician-diagnosed CD cases surveyed (5 vs. 46). Although there is poor availability of gluten-free products in Brazil and the cost of these products is high compared to their regular counterparts [32], the general prevalence of adherence to a GFD (7.48%) in the population studied is the highest among the prevalence rates reported in other surveys carried out in Latin America. Particularly, this prevalence rate is two-fold higher than the rate reported in a survey carried out in Mexico (3.7%) [13], the high cost and poor availability of gluten-free products have been documented in Mexico [33]. This highlights that there is not enough data to establish a clear association between the cost and availability of gluten-free products and the prevalence rates of adherence to a GFD, at least in Latin America. Younger age at the time of diagnosis and longer duration of disease, among others, are factors associated with poor adherence to a GFD in CD cases [34]. Similarly, our results show that in the absence of a formally diagnosed GRD those aged ≥39 years old more frequently follow a GFD. Certainly, the motivations for adhering to the diet without a diagnosis of a GRD commonly include weigh control or a perceived general health benefit [9,10,35,36]. In the present study, the non-SR-GS individuals commonly reported those motivations for following a GFD or avoiding wheat/gluten from their diets although there is not enough evidence to support health benefits of the GFD in the absence of GRD [37] and the benefits and risks of following a GFD for this group of people are uncertain [12]. Overall, most people who were following a GFD were doing it for reasons other than the treatment of a diagnosed GRD and 50% of the GFD cases were following the diet without medical/dietitian advice. 

## 5. Conclusions

Based on data about the proportion of CD cases reported, wheat/gluten-induced symptoms frequency and adherence to a GFD, GRD are underdiagnosed in Brazilian population although the prevalence of adherence to a GFD is the highest among the Latin American populations studied. The results also add to the growing body of evidence for highlighting the underdiagnosis of GRD and the trend for following a GFD without scientific evidence of health benefits in the absence of GRD in the Latin American region.

## Supplementary Materials

The following are available online at https://www.mdpi.com/1010-660X/56/4/163/s1, Table S1: Definitions and self-reported prevalence rates of adverse reactions to food and GRDs., Supplementary Material S2: English Version of the Questionnaire.
