# Supplementary material for: Prevalence of Adverse Reactions to Glutenand People Going on a Gluten-Free Diet:A Survey Study Conducted in Brazil
Source: Medicina (Kaunas). 2020 Apr 4;56(4):163. doi: 10.3390/medicina56040163 (PMC7231032; doi:10.3390/medicina56040163)
Supplement: Supplementary file 1 [file medicina-56-00163-s001.pdf]

Supplementary material.
Table S1. Definitions and self-reported prevalence rates of adverse reactions to food and GRDs.

**Condition**	**Definitions**
Adverse reaction to foods	Subjects who reported that the food-induced symptoms occurred always or most of the time (recurrent) or sometimes (non-recurrent).
Adverse reaction to gluten	Subjects who reported that the gluten-induced symptoms occurred always or most of the time (recurrent) or sometimes (non-recurrent).
SR-GS*	Subjects who met criteria for recurrent adverse reaction to wheat/gluten and were also following a GFD*.
SR-PD CD	Subjects who reported that a physician or a health professional diagnosed them celiac disease.
NCGS	SR-PD NCGS* Subjects who reported that a physician diagnosed them.
	SR-NCGS* Subjects who met the following: (1) individuals who did not meet criteria for self-reported Physician diagnosed CD or wheat allergy; (2) individuals who did not meet criteria for self-reported wheat allergy; and (3) individuals who met criteria for SR-GS*.
Wheat allergy	SR-PD WA*Subjects who reported that a physician diagnosed them.
	SR-WA* Subjects who reported recurrent adverse reactions convincing of food allergy.

Acronyms used: SR-GS: Self-Reported Gluten Sensitivity; SR-PD: Self-Reported Physician-Diagnosed; NCGS: Non-Celiac Gluten Sensitivity; GFD: Gluten-Free Diet.

S2. English Version of the Questionnaire

Please indicate with an X the answer to each of the following questions

1. Do you have some discomfort or adverse reaction when consuming wheat products?

Yes (Go to question 2, please answer only section 1)
No (Go to question 20, please answer only section 2)

ID:_________ Full Name:_______________________________________Age:_______Date of birth: / / Female Male Level of education:_______________

Home Phone:________________Cell/mobile:__________________Email:_____________________

SECTION 1

2. Have you ever been diagnosed with a disease related to the consumption of wheat or gluten?

Yes (which of the following?) No **(go to question 4)**

a) Wheat allergy
b) Dermatitis herpetiformis
c) Coeliac disease
d) Wheat or gluten ataxia
e) Sensitivity or intolerance to wheat
f) Other (Which one?)

3. Who made the diagnosis?

a) Physician or General Practitioner (GP)
b) Gastroenterologist
c) Homeopath
d) Chemist
e) Dietitian
f) Nutritionist
g) Other, who?

4. What kind of discomfort or gastrointestinal symptoms have you shown repeatedly by consuming foods with wheat or gluten?

a) Stomach pain
b) Diarrhea
c) Vomiting
d) Nausea
e) Reflux

f) Upset stomach
g) Bloating
h) Abdominal bloating
i) Constipation
j) Acidity
k) Other (Which one?)
l) None (Please go to question 7)

5. When you eat food with wheat or gluten, how often do the gastrointestinal symptoms mentioned above occur?

a) Always
b) Most of the time
c) Occasionally

6. After eating foods with wheat or gluten, how long does it take for the gastrointestinal symptoms referred to above to appear?

a) 10 min or less
b) 10 min–1 h
c) 1–2 h
d) In the first 6 h
e) Between 6 h and 24 h
f) After 24 h
g) Do not know

7. What kind of discomfort or no bowel symptoms have you had repeatedly by consuming foods with wheat or gluten?

a) Hives or rashes that itch
b) Difficulty breathing
c) Swelling of face or lips
d) Fatigue
e) Muscle pain
f) Pain in the joints
g) Lack of Wellbeing
h) Rhinitis
i) Dermatitis
j) Anxiety
k) Confusion
l) Headache
m) Numbness
n) Depression
o) Other (Which one?)
p) None **(If you choose this option, please go to question 10)**

8. When you eat foods with wheat or gluten, how often the non-intestinal discomfort or symptoms mentioned above occur?

a) Always
b) Most of the times
c) Occasionally

9. After eating foods with wheat or gluten, how long it takes for discomfort referred above to appear?

a) 10 min or less
b) 10 min–1 h
c) 1–2 h
d) In the first 6 h
e) Between 6 and 24 h
f) After 24 h
g) Do not know

10. Have you been diagnosed with any of the following?

a) Irritable bowel syndrome
b) Lactose intolerance
c) Intolerance to food other than lactose (which one?)
d) Allergy (Which one?)
e) Any type of psychiatric disorder?
f) Gastrointestinal cancer
g) Eating disorder (which one?)
h) Autoimmune disease such as diabetes mellitus.
i) Colitis
j) None of the previous diseases

11. Does anyone in your family have been diagnosed with coeliac disease?

a) Yes
b) No
c) Do not know

12. Do you keep a diet free of wheat or gluten?

a) Yes **(go to the next question)**
b) No **(go to question 15)**

13. Who instructs the diet free of wheat or gluten?

a) Nutritionist
b) Dietitian
c) Physician or General practitioner
d) Gastroenterologist
e) Chemist
f) Homeopath
g) Yourself
h) Other, who?

14. What are the main motivations for keeping a diet free of wheat or gluten?

a) Weight control
b) Having a relative with celiac disease or wheat/gluten sensitivity or intolerance
c) The gluten-free products taste better
d) A diet free of wheat or gluten is healthier
e) The symptoms triggered after gluten ingestion
f) Other reason, which one?

(Stop)

15. Do you avoid, as much as possible, wheat- or gluten-based products from your diet?

a) Yes **(Go to the next question)**
b) No **(Stop)**

16. What are the main motivations for avoiding, as much as possible, wheat or gluten from your diet?

a) The symptoms triggered after gluten ingestion
b) Weight control
c) Having a relative with celiac disease or wheat/gluten sensitivity or intolerance
d) The gluten-free products taste better
e) Avoiding wheat or gluten is healthy
f) Other reason, which one?

Thank you very much!

SECTION 2

17. Do you have some discomfort or adverse reaction when consuming a particular food?

a) Yes (Go to next question)
b) No (Please go to question 20)

18. Have you been diagnosed with any of the following diseases?

a) Lactose intolerance
b) Intolerance to food other than lactose (Which one?)
c) Any type of allergy
d) Any type of psychiatricdisorder
e) Gastrointestinal cancer
f) Eating disorder (Which one?)
g) Autoimmune disease such as diabetes mellitus.
h) Colitis
i) Irritable Bowel Syndrome
j) None of the previous diseases
